# Efficacy and safety of antithrombotic therapy for preventing and treating pediatric thromboembolic disease: a systematic review

**DOI:** 10.1038/s41598-024-64334-8

**Published:** 2024-06-11

**Authors:** Hongjin Gao, Mingyu Chen, Youqi Huang, Huiting Liu, Yuze Lin, Min Chen

**Affiliations:** 1 Department of Pharmacy, Fujian Provincial Hospital, Fuzhou, 350001 China; 2 School of Pharmacy, Fujian Medical University, Fuzhou, 350004 China; 3 Shengli Clinical College of Fujian Medical University, Fujian Provincial Hospital, No.134 Dongjie St., Fuzhou, 350001 China

**Keywords:** Drug safety, Paediatric research

## Abstract

This review used traditional and network meta-analyses (NMA) to conduct a comprehensive study of antithrombotic therapies in children with thromboembolic disease. We searched the PubMed, Embase, Cochrane Library, Web of Science and ClinicalTrials.gov databases from their inception to 26 February, 2023. And we finally included 16 randomized controlled trials. In the prevention of thromboembolic events (TEs), the use of anticoagulants had a low risk of TEs (relative risk (RR) 0.73, 95% CI 0.56 to 0.94) and a high risk of minor bleeding (RR 1.43, 95% CI 1.09 to 1.86) compared with no anticoagulants. In the treatment of TEs, direct oral anticoagulants (DOACs) were not inferior to standard anticoagulation in terms of efficacy and safety outcomes. In NMA, rivaroxaban and apixaban showed the lowest risk for TEs and major or clinically relevant nonmajor bleeding. According to the overall assessment of efficacy and safety, dabigatran may be the best choice for children with thromboembolic disease. The results of our study will provide references and suggestions for clinical drug selection.

## Introduction

Along with the development of high-resolution imaging modalities and increased awareness of childhood thromboembolic events (TEs), the incidence of pediatric TEs is rapidly increasing^1^. 95% of Venous thromboembolic events (VTEs) in children are secondary to serious diseases such as cancer, nephrotic syndrome, trauma/surgery, congenital heart disease, systemic lupus erythematosus and so on. In addition, central venous lines (CVLs) used for short-term intensive care, hemodialysis or long-term supportive care in patients with serious disease are the most common risk factor for VTEs^2^. Although the number of relevant studies on pediatric thromboembolic disease is gradually increasing, the evidence is still relatively scant compared with adults, and much evidence has been inferred from adult practice. It is worth noting that differences in the anatomical distribution, the pathophysiology of thrombosis, and the coagulation system between adults and children impact the effects of antithrombotic drugs in vivo^3,4^. Thus, there is an urgent need for more specialized studies on thromboembolic disease in children and for a summary of the existing evidence to provide a reference for future clinical research.

Regarding anticoagulant therapy in children, the relevant guidelines favor standard therapy, including unfractionated heparin (UFH), low-molecular-weight heparin (LMWH), fondaparinux, and vitamin K antagonists (VKAs), as these drugs are already used in children, and there are published safety data for each^3^. However, direct oral anticoagulants (DOACs) (e.g. dabigatran, rivaroxaban, apixaban and edoxaban), which have been used in adult antithrombotic indications, have a potential advantage over standard anticoagulant therapy. This includes more consistent pharmacokinetics, that do not require frequent laboratory monitoring, and showed reduced risk for intravenous administration, improved patient compliance, and has acceptable efficacy and safety^5^. Recently, rivaroxaban and dabigatran with age-appropriate formulations have been approved in some areas to treat and prevent venous thromboembolism (VTE) in children^6^. However, there are still some concerns regarding its clinical application. Some randomized controlled trials (RCTs) have reported that the risk for TEs in children treated with DOACs appears to be less than standard anticoagulation (SAC), but the risk for adverse events is higher, particularly in terms of major bleeding and clinically related nonmajor bleeding^7–9^. Due to their incomplete physical development, children are more likely to have TEs which are difficult to detect. Their ability to bear the risk for adverse reactions is poorer than it is for adults^4^. Until now, there is still no consensus on the best antithrombotic therapy for preventing or treating thromboembolic diseases in children.

In order to comprehensively summarize the available clinical evidence, we first performed a systematic review and meta-analysis to study the efficacy and safety of prophylactic and therapeutic anticoagulation, respectively, in broader pediatric population with thromboembolic disease. Furthermore, we compared all antithrombotic regimens using network meta-analysis (NMA) to investigate the optimal intervention.

## Methods

This systematic review using traditional meta-analysis and NMA was conducted according to the Preferred Reporting Items for Systematic Reviews and Meta-Analyses (PRISMA) (Supplementary Table S1) and the PRISMA Extension Statement for Reporting of Systematic Reviews Incorporating Network Meta-analyses of Health Care Interventions (PRISMA-NMA) (Supplementary Table S2)^10^. We published the protocol of this review in PROSPERO (CRD42023455365).

### Data sources and searches

We searched relevant articles in the PubMed, Embase, Web of Science and Cochrane Library databases without restriction until 26 February, 2023. The detailed search formulas we used are presented in Supplementary Table S3. For comprehensiveness, we manually searched the references cited by relevant articles and screened for unpublished RCTs with results in ClinicalTrials.gov. Two researchers (Mingyu Chen and H.G.) searched and screened the literature separately. Studies comparing DOACs, SAC, antiplatelet drugs and other no anticoagulant therapies were included. SAC therapy comprises of UFH, LMWH, VKAs and synthetic pentasaccharide Xa inhibitors.

### Inclusion and exclusion criteria

The eligibility criteria were drafted according to PICOS (Population, Intervention, Comparison, Outcome, Study design) statement by two researchers (Mingyu Chen and Y.H.) and revised by another (H.G.).

The inclusion criteria were: (P) patients aged ≤ 18 years undergoing antithrombotic therapy and receiving drugs at least 4 weeks regardless of the purpose of medication; (I/C) with efficacy or safety outcomes in the comparison among DOACs, SAC (UFH, LMWH, VKAs, and synthetic pentasaccharide Xa inhibitors), antiplatelet drugs, and no anticoagulants, including anticoagulants below the prophylactic dose to flush catheters, and usual care with or without placebo; (O) efficacy outcomes, including TEs and repeat imaging tests compared with baseline and results that were diagnosed as normalized, and improved; (O) safety outcomes, including major bleeding, the composite of major and clinically relevant nonmajor bleeding (CRB), minor bleeding and all-cause mortality; (S) RCTs.

The exclusion criteria were: (1) studies with duplicate or incomplete data; (2) patients diagnosed with congenital bleeding or thrombotic disease.

### Outcomes measures

The primary efficacy outcome was TEs (venous or arterial), defined as the appearance of a new or recurrent thrombotic burden within the cardiovascular system noted on either routine surveillance or clinically indicated imaging, and the occurrence of a clinical event known to be strongly associated with thrombus (e.g. stroke or pulmonary embolism). The secondary efficacy outcome was the results of repeat imaging tests.

The primary safety outcome was major bleeding, as defined by the International Society on Thrombosis and Haemostasis. Secondary safety outcomes included CRB, minor bleeding and all-cause mortality. The definitions of efficacy and safety outcomes were consistent across all trials, shown in Supplementary Table S4.

### Data extraction and risk of bias assessment

Two blinded authors (Y.H. and H.L.) independently extracted the following data from the included studies: study design, authors and publication information, population information (number of patients, mean or median age, sex ratio, and relevant diseases), antithrombotic regimen, study period, data of efficacy and safety outcomes. Researchers resolved disputes through discussion, and a final resolution was provided by H.G..

Two independent researchers (Y.H. and Y.L.) used Cochrane Collaboration’s tool (Risk of Bias 2) to assess the risk of bias, including randomization process, deviations from intended interventions, missing outcome data, measurement of the outcome, and selection of the reported result^11^. Each item was evaluated as showing low, high, or some concerns. During the assessment, disputes were resolved by H.G..

### Statistical analyses

We classified the identified studies into prevention or treatment studies based on the purpose of medication, and conducted a systematic review and meta-analysis to study the efficacy and safety of prophylactic and therapeutic anticoagulation, respectively. We calculated the results using relative risk (*RR*) and 95% confidence interval (95% CI). We adopted the *I*^2^-index to identify heterogeneity, where *I*^2^ > 50%, the random-effects model was used; otherwise, the fixed-effects model was used^12^. Funnel plots were conducted to assess publication bias by measuring efficacy and safety outcomes.

We also used fully Bayesian, arm-based, random-effects NMA to compare TEs and CRB among antithrombotic therapies in children with thromboembolic diseases. NMA combines direct evidence (direct comparison) with indirect evidence (comparison through other interventions) to improve the accuracy of estimates^13–15^. We used the ordinary consistency model^16^ and the likelihood binomial/log model to evaluate the comparability of direct and indirect evidence^17^. The unrelated mean effects (UME) model was used for evaluating the global consistency assumption in the NMA by compare the data of deviance information criterion (DIC)^18^. And we also used the node-splitting method for assessment of local inconsistencies, there was no inconsistency when *P* > 0.05^19^. To evaluate the similarity among studies, we collected the relevant descriptive statistics and compared them based on the PICO methodology^19–21^. We assessed heterogeneity by calculating the *I*^2^-index across all included studies.

The inference was performed using means and 95% CI, and it drew from the marginal posterior distribution of a Monte Carlo Markov chain (MCMC) with 50,000 iterations. Model convergence was assessed by analyzing history, running means density and Brooks-Gelman-Rubin diagnostic plots^19^. Rank probabilities were plotted based on the surface under the cumulative ranking curves (SUCRA) for all effective interventions, including four DOACs, SAC and aspirin.

All statistical analyses were performed using R 4.2.2 and RevMan version 5.3. Outcomes are expressed as *RR* with 95% CI.

## Results

**Figure 1:**
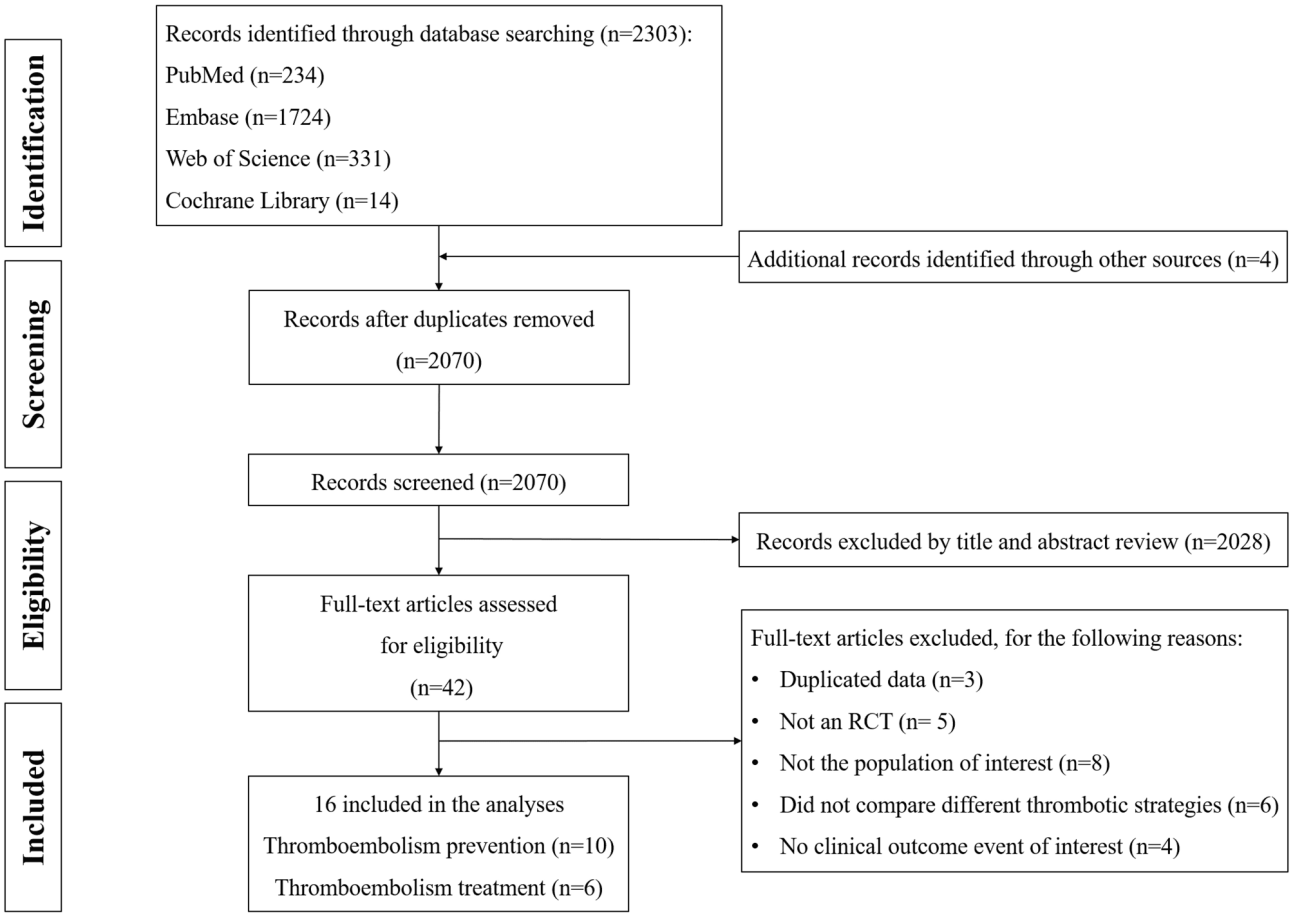
Flowchart of the literature search and the study selection process.

We identified 2303 studies with our search strategy, of which 16 were included in our final analyses (Fig. 1)^7,8,22–32^. Among them, 3 completed trials (NCT02369653, NCT02981472 and NCT02798471) have not yet been published in peer-reviewed journals, data were derived from the results recorded on ClinicalTrials.gov. The overall population consisted of 2916 patients with thromboembolic disease, 1098 on DOACs, 993 on SAC, 719 on no anticoagulants and 106 on aspirin. Male sex ranged from 43.5 to 65.3% and the study period ranged from 28 days to 2 years. The 16 RCTs included were open-label. Ten of RCTs involved the prevention of any thromboembolism, and six involved the treatment of VTE. Detailed characteristics and information for all of the studies were given in Table 1 and Supplementary Table S5.

**Table 1:** Baseline characteristics of the studies included in the meta-analysis.

Author, year	Study design	Population	Purpose	Sample size	Antithrombotic regimen^a^	Study period	Mean or median age	Male (%)	Outcome^b^
Massicotte P et al., 2003 ^22^	RCT, Open-Label	Children with new CVLs	P	186	SAC (LMWH1) vs H	1 m	6.1 y/6.4 y	97 (52.2%)	1,3,4,5,6
Ruud E et al., 2006 ^23^	RCT, Open-Label	Children with malignancies placed new CVLs	P	62	SAC (VKA1) vs U	6 m	7.3 y/6.2 y	31 (50%)	1,3,4
NCT02369653, 2015	RCT, Open-Label	Children with ALL or lymphoma placed CVCs	p	512	A vs U	40d	7.2 y/7.1y	290 (56.6%)	1,3,4,5,6
NCT02981472, 2016	RCT, Open-Label	Children with congenital or acquired heart disease	p	192	A vs SAC	1y	8.0 y/7.6 y	102 (53.1%)	3,4,5
Greiner J et al., 2019 ^24^	RCT, Open-Label	Children with ALL placed new CVLs	P	949	SAC (LMWH2) vs AT vs H	33–64 d	Comparable age groups	537 (56.6%)	1,3,4
Faustino EVS et al., 2021 ^25^	RCT, Open-Label	Children with new CVCs	P	51	SAC (LMWH2) vs U	28 d	2.8 y/1.0 y	23 (45.1%)	1,4,5,6
Portman MA et al., 2022 ^26^	RCT, Open-Label	Children with cardiac diseases	P	167	E vs SAC	1 m	8.2 y/7.8 y	109 (65.3%)	1,4,5
Monagle P et al., 2011 ^27^	RCT, Open-Label	Children with Fontan procedure	P	111	SAC (VKA2) vs ASA1	2 y	5.1 y/4.6 y	71 (64.0%)	1,3,4,5,6
Pessotti CF et al., 2014 ^28^	RCT, Open-Label	Children with Fontan procedure	P	30	SAC (VKA2) vs ASA2	2 y	5.8 y/4.8 y	17 (56.7%)	1,6
McCrindle BW et al., 2021 ^29^	RCT, Open-Label	Children with Fontan procedure	P	100	R vs ASA1	1 y	4.1 y/4.2 y	59 (59%)	1,3,4,5
NCT02798471, 2016	RCT, Open-Label	Children with VTE	T	290	E vs SAC	3 m	10.9 y/11.1 y	150 (51.7%)	1,4,6
Thom K et al., 2020 ^30^	RCT, Open-Label	Children with CVC-VTE	T	126	R vs SAC	1–3 m	Comparable age groups	70 (56%)	1,2,4
Connor P et al., 2020 ^31^	RCT, Open-Label	Children with CVT	T	114	R vs SAC	3 m	Comparable age groups	69 (60.5%)	1,2,3,4
Eghbali A et al., 2020 ^8^	RCT, Open-Label	Children with DVT or PE	T	23	D vs SAC	6 m	12.1 y/11.4 y	10 (43.5%)	1,3,4,5
Halton J et al., 2021 ^7^	RCT, Open-Label	Children with VTE	T	267	D vs SAC	85 d	11.1 y/11.0 y	133 (50.0%)	1,2,3,4,5,6
Palumbo JS et al., 2022 ^32^	RCT, Open-Label	Children with active cancer–associated VTE	T	56	R vs SAC	3 m	Comparable age groups	31 (55.4%)	2,4,6

*P* prevention, *T* treatment, *CVLs* central venous lines, *CVCs* central venous catheters, *SP* synthetic pentasaccharide, *UFH* unfractionated heparin, *LMWH* low-molecular-weight heparin, *VKA* vitamin K antagonist, *VTE* venous thromboembolism, *DVT* deep vein thrombosis, *PE* pulmonary embolism, *CVT* cerebral venous thrombosis, *ALL* acute lymphoblastic leukaemia, *INR* international normalized ratio, *y* years, *m* months, *d* days.

^a^Antithrombotic regimen: *LMWH1* Reviparin-sodium (< 3 months-50 IU/kg; ≥ 3 months-30–50 IU/kg) twice daily, *LMWH2* Enoxaparin (≤ 2 months–0.75 mg/kg; > 2 months to 0.4–0.5 mg/kg) Once every 12 h, *H* heparin to flush catheters (< 3 IU/kg/h), below the prophylactic dose, *U* usual care with or without placebo, *VKA1* VKAs with target INR between 1.3 and 1.9, *VKA2* VKAs with target INR between 2.0 and 3.0, *AT* Antithrombin(weight-based dose), *A* Apixaban (body weight–adjusted 2–4 mg) twice daily, *R* Rivaroxaban (body weight–adjusted 20 mg equivalent doses) once daily, *E* Edoxaban (age and weight-based dose) once daily, *D* Dabigatran etexilate (age and weight-based dose) twice daily, *ASA1* aspirin (5 mg/kg) once daily, *ASA2* aspirin (10 mg/kg) once daily, *SAC* Standard anticoagulation comprised LMWHs, UFH, VKAs, or SP Xa inhibitors, used according to investigators’ judgment and standard clinical practice, at Prophylactic or therapeutic dose.

^b^Outcome: 1: represents thromboembolic events. 2: represents repeat imaging outcomes. 3: represents major bleeding. 4: represents major or clinically relevant nonmajor bleeding. 5: represents minor bleeding. 6: represents all-cause mortality.

In order to reduce the differences between studies, we included 5 studies in the prevention group for the meta-analysis of the comparison between anticoagulants (DOACs/SAC) and no anticoagulants. Studies on the comparison between DOACs and SAC (Portman et al., 2022 and NCT02981472, 2016), and the comparison between anticoagulants and aspirin (Monagle et al., 2011, Pessotti et al., 2014 and McCrindle et al., 2021) were excluded. In addition, we included all studies in the treatment group for the meta-analysis of the comparison between DOACs and SAC. Finally, we conducted the NMA of TEs and CRB in all antithrombotic therapies.

### Risk of bias assessment

According to the Risk of Bias 2 evaluation, 81.3% of the included studies were considered at low risk of bias, while 18.8% demonstrated some concerns for intention-to-treat analysis. Source of some concerns include the domains of randomization process, deviations from intended interventions and measurement of the outcome. The risk of bias of the 16 studies included in our research was reasonable (Supplementary Figs. S1 and S2).

### Publication bias

For the comparison of 5 studies in the prevention group and 6 studies in the treatment group, visual inspection showed a publication bias for TEs and major bleeding according to Begg’s funnel plots in prevention group, and for TEs and CRB in treatment group, while the other outcomes were visually symmetrical (Supplementary Figs. S9 and S10).

### Similarity assumption

In the similarity assumption, we assessed population characteristics, study period, efficacy and safety outcomes definition of the included studies. Taking into account the distinction of population, the RCTs were classified in terms of prevention and treatment for the meta-analysis based on the purpose of intervention.

For NMA of efficacy, due to the distinction of population and efficacy outcome (TEs), the RCTs were also classified for prevention and treatment. Considering the similarity of intervention, comparison and safety outcome (CRB) in RCTs and the few studies of pediatric thromboprophylaxis, we conducted an NMA of CRB in studies covering both prevention and therapeutic population and carefully assessed the heterogeneity among included studies.

### Meta-analysis of prevention group

**Figure 2:**
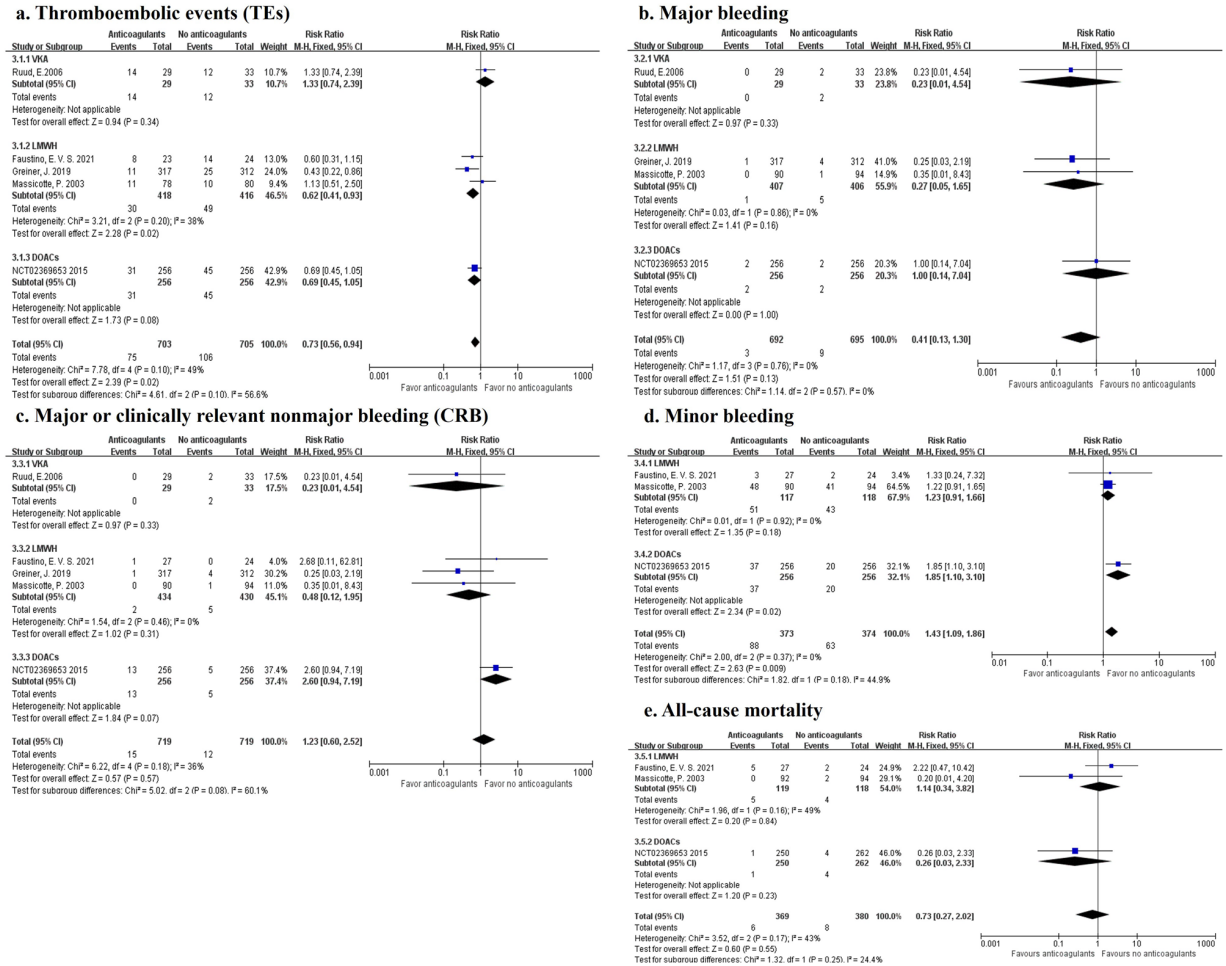
Forest plot showing the efficacy and safety outcomes between patients taking anticoagulants and those taking no anticoagulants in the prevention group. Note: *VKA* vitamin K antagonist, *LMWH* low-molecular-weight heparin, *DOACs* direct oral anticoagulants.

*Efficacy outcomes.* We conducted a meta-analysis of the included prevention group studies between anticoagulants (DOACs/SAC) and no anticoagulants, and we set up subgroups according to the type of anticoagulant therapy. There were 5 studies reported the outcomes of TEs (75 events in 703 patients during anticoagulant prevention and 106 events in 705 patients during no anticoagulant prevention). The combined data showed that the use of anticoagulants could reduce the risk for TEs compared to using no anticoagulants (*RR* 0.73, 95% CI 0.56 to 0.94, *I*^2^ = 49%). A subgroup analysis of LMWH showed a significantly low risk for TEs (*RR* 0.62, 95% CI 0.41 to 0.93, *I*^2^ = 38%). There were no differences across other subgroups (Fig. 2a).

*Safety outcomes.* A total of 4 studies recorded data on major bleeding (3 events in 692 patients during anticoagulant prevention and 9 events in 695 patients during no anticoagulant prevention). No statistical significance was found in combined data or subgroup data (*RR* 0.41, 95% CI 0.13 to 1.30, *I*^2^ = 0%) (Fig. 2b). CRB was evaluated in 5 studies (15 in 719 patients using anticoagulants and 12 in 719 controls). There were no statistical significances in combined data or subgroup data (*RR* 1.23, 95% CI 0.60 to 2.52, *I*^2^ = 36%) (Fig. 2c). For the outcome of minor bleeding (88 in 373 patients using anticoagulants and 63 in 374 controls), it was found that patients treated with anticoagulants could have a greater risk for minor bleeding relative to those treated with no anticoagulants (*RR* 1.43, 95% CI 1.09 to 1.86, *I*^2^ = 0%) (Fig. 2d).

All-cause mortality was evaluated in 3 studies (6 in 369 patients using anticoagulants and 8 in 380 controls). No statistical significance was found in combined data or subgroup data (*RR* 0.73, 95% CI 0.27 to 2.02, *I*^2^ = 43%) (Fig. 2e).

### Meta-analysis of treatment group

**Figure 3:**
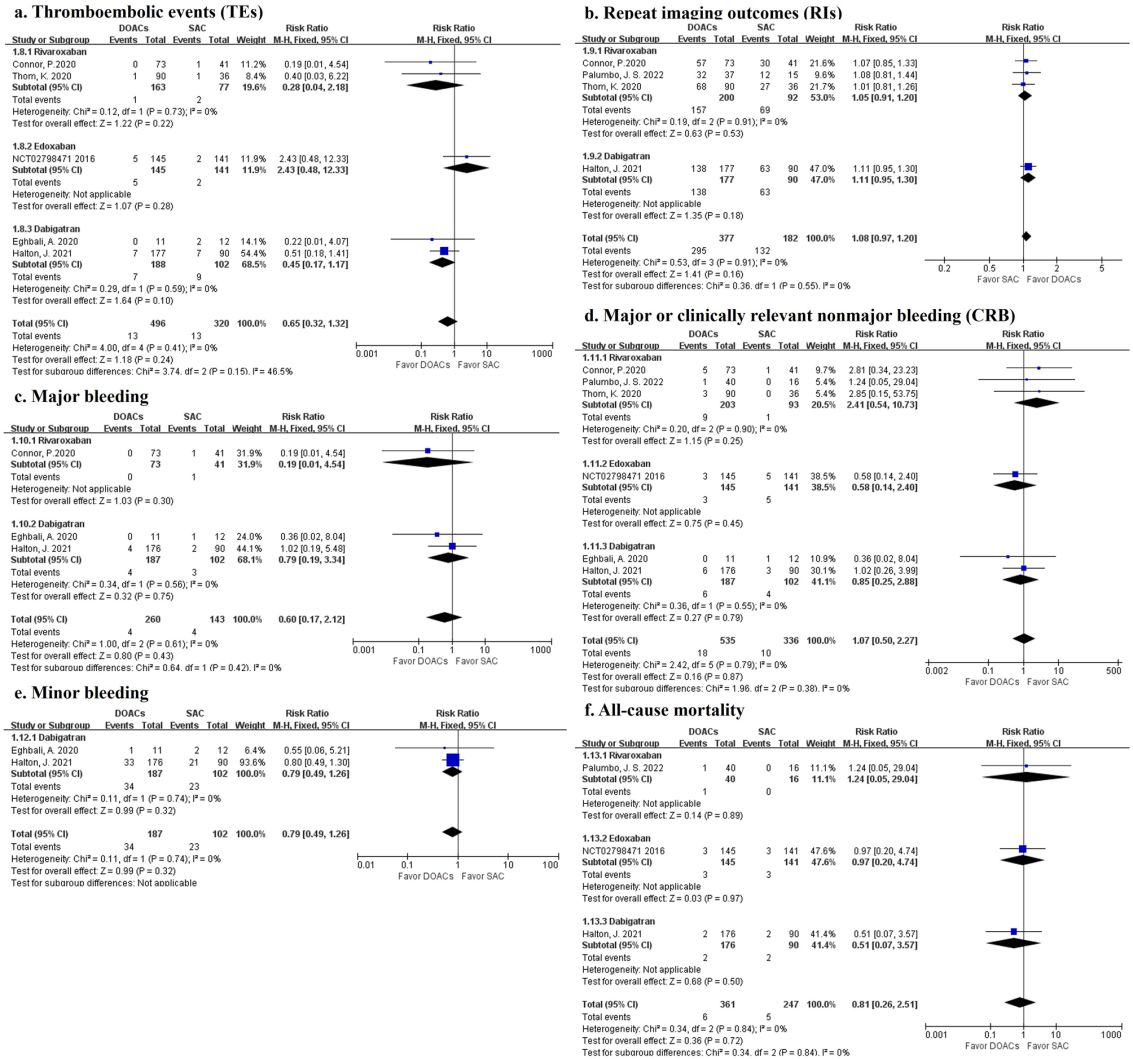
Forest plot showing the efficacy and safety outcomes between patients taking DOACs and those taking SAC in the treatment group. *Note: DOACs* direct oral anticoagulants, *SAC* standard anticoagulation.

*Efficacy outcomes.* We conducted a meta-analysis of the included treatment group studies between DOACs and SAC, and we set up subgroups according to the type of DOACs. No statistical significance was found in RCTs for the outcome of TEs (13 in 496 patients receiving DOACs and 13 in 320 receiving SAC), but DOACs had a relative low risk for TEs compared to SAC (*RR* 0.65, 95% CI 0.32 to 1.32, *I*^2^ = 0%) (Fig. 3a). Repeat imaging outcomes that were compared to baseline showed the same results (295 in 377 patients used DOACs and 132 in 182 controls) (*RR* 1.08, 95% CI 0.97 to 1.20, *I*^2^ = 0%) (Fig. 3b). There were no differences found in subgroup analysis.

*Safety outcomes.* A total of 3 studies recorded data on major bleeding (4 in 260 patients using DOACs and 4 in 143 using SAC) and 2 studies did on minor bleeding (34 in 187 patients with DOACs and 23 in 102 controls). No statistical significance was found in combined data or subgroup data, but DOACs had a relative low risk for major and minor bleeding compared to SAC (major bleeding, *RR* 0.60, 95% CI 0.17 to 2.12, *I*^2^ = 0%; minor bleeding, *RR* 0.79, 95% CI 0.49 to 1.26, *I*^2^ = 0%) (Fig. 3c,e). CRB was evaluated in 6 studies (18 in 535 patients with DOACs and 10 in 336 controls). There were no differences in CRB between DOACs and SAC (*RR* 1.07, 95% CI 0.50 to 2.27, *I*^2^ = 0%) (Fig. 3d) and in the outcomes of all-cause mortality (*RR* 0.81, 95% CI 0.26 to 2.51, *I*^2^ = 0%) (Fig. 3f).

### NMA among antithrombotic therapies

*Efficacy outcome (TEs).* We conducted a NMA of TEs among the prevention group studies. Considering the few studies of pediatric thromboprophylaxis, we combined the DOACs for the NMA in prevention group. A total of 403 patients from 4 RCTs were included, 173 (42.93%) took DOACs, 125 (31.02%) used SAC, 105 (26.05%) took aspirin. The network plot graphically represents the evidence base (Supplementary Fig. S3a). In the rank probabilities of TEs (Supplementary Fig. S5a), DOACs had the lowest risk for TEs, followed by SAC and aspirin. However, there were no significant differences. The NMA results for TEs are shown in forest plot and head-to-head comparisons table (Supplementary Fig. S4a and Supplementary Table S6a).

The DIC of consistency model and UME model were similar (consistency model, DIC = 15.3673; UME model, DIC = 14.8658). No inconsistency was revealed between DOACs versus aspirin (*P* = 0.15), DOACs versus SAC (*P* = 0.14) and SAC versus aspirin (*P* = 0.16). There was no significant heterogeneity, with a global *I*^2^ of 14.0%. But some heterogeneity was revealed in pair-wise comparisons (Supplementary Fig. S6).

In addition, we conducted a NMA of TEs among the treatment group studies. 816 patients from 5 RCTs were included, 320 (39.22%) took SAC, 188 (23.04%) used dabigatran, 163 (19.98%) took rivaroxaban, and 145 (17.77%) used edoxaban. The network plot graphically represents the evidence base (Supplementary Fig. S3b). Comparisons between 3 DOACs and SAC showed that dabigatran and rivaroxaban had a relative low risk for TEs, and edoxaban had a relative high risk for TEs (Supplementary Fig. S4b). However, there were no significant differences. The other NMA results for TEs are shown in head-to-head comparisons (Supplementary Table S6b).

In the rank probabilities of TEs (Supplementary Fig. S5b), rivaroxaban had the lowest risk for TEs, followed by dabigatran, SAC, and edoxaban. The DIC of consistency model and UME model were similar (consistency model, DIC = 19.3190; UME model, DIC = 19.2162), and the NMA was basically consistent with consistency assumption globally. We could not assess local inconsistency due to the absence of closed loops in the network. No significant heterogeneity was revealed, with a global *I*^2^ of 15.0%. But some heterogeneity was revealed in pair-wise comparisons (Supplementary Fig. S7).

**Figure 4:**
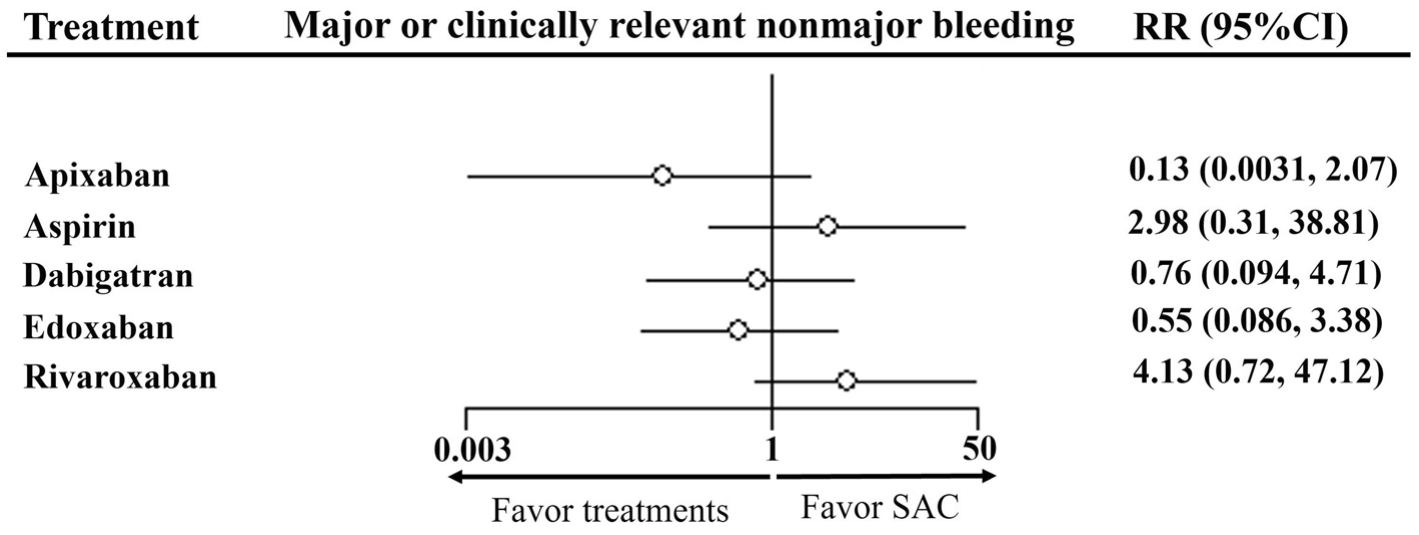
Forest plot showing the safety outcome (CRB) of DOACs versus SAC in NMA of RCTs. *Note*: *CRB* major or clinically relevant nonmajor bleeding, *NMA* network meta-analysis, *RCTs* randomized controlled trials, *RR* risk ratio, CI confidence interval, *SAC* standard anticoagulation.

**Figure 5:**
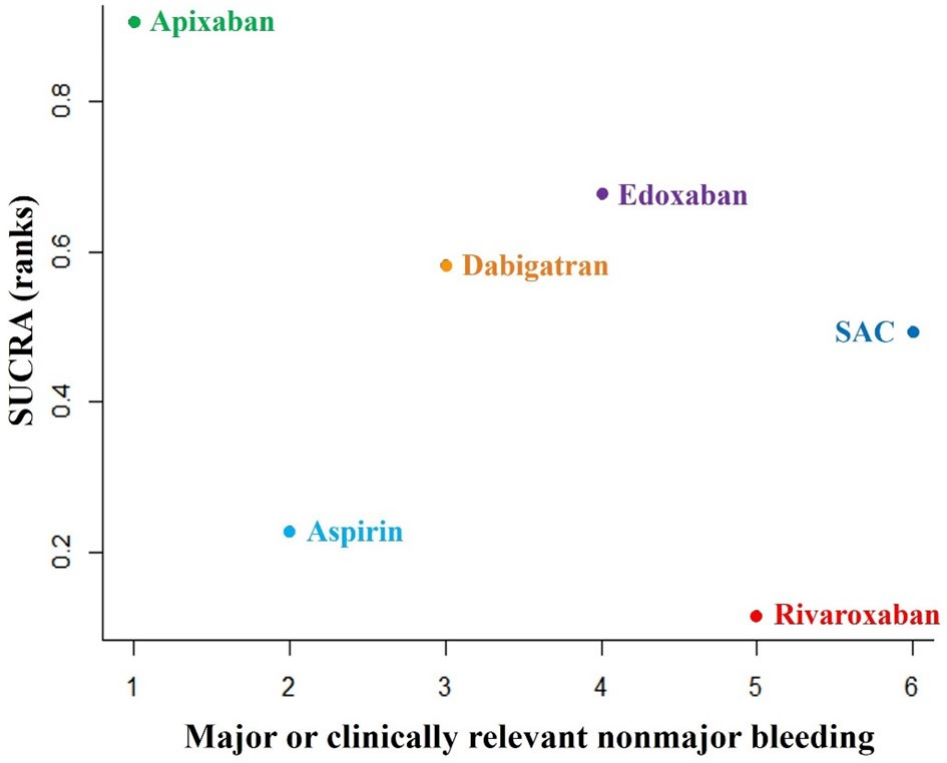
Ranking plot for major or clinically relevant nonmajor bleeding. Note: *SAC* standard anticoagulation, *SUCRA* surface under the cumulative ranking curves. Higher SUCRA number indicates lower risk of events.

*Safety outcome (CRB).* Overall, 1435 patients from the 10 RCTs were included, 510 (35.54%) took SAC, 267 (18.61%) used rivaroxaban, 187 (13.03%) took dabigatran, 254 (17.70%) used edoxaban, 126 (8.78%) took apixaban and 91 (6.34%) used aspirin. A network plot map represents the evidence base (Supplementary Fig. S3c). The comparisons between other antithrombotic therapies and SAC showed that apixaban, dabigatran, and edoxaban had a relative low risk for CRB, whereas aspirin and rivaroxaban had a relative high risk (Fig. 4). The other NMA results for CRB are shown in head-to-head comparisons (Supplementary Table S6c). In the rank probabilities of CRB (Fig. 5), apixaban had the lowest risk for CRB, followed by edoxaban, dabigatran, SAC, aspirin, and rivaroxaban. There were significant differences in apixaban and rivaroxaban (*RR* 0.03, 95% CI 0 to 0.81).

These results of consistency model and UME model were similar (consistency model, DIC = 35.7257; UME model, DIC = 37.2903), and no inconsistency was revealed between rivaroxaban versus aspirin (*P* = 0.37), SAC versus aspirin (*P* = 0.38) and SAC versus rivaroxaban (*P* = 0.37). There was no significant heterogeneity in global assessment and pair-wise comparisons, with a global *I*^2^ of 0.4% (Supplementary Fig. S8).

## Discussion

At present, for thromboembolic disease in children, much of the evidence regarding the use of antithrombotic therapy is inferred from adult practice^3^. This review comprehensively outlines available clinical evidence of optimal antithrombotic strategies for children with such disease. In a meta-analysis, we separately compared the efficacy and safety outcomes of therapies for preventing and treating thromboembolic disease. In addition, we conducted the NMA of TEs and CRB in antithrombotic therapies. Through the further analysis of high-quality evidence, we sought to fully reflect the overall situation for the application of antithrombotic therapies in children. We also provide references and suggestions for clinical decision-making.

### Meta-analysis of prevention group

In our study, patients receiving anticoagulants were at reduced risk for TEs compared to those taking no anticoagulants in prevention group, particularly with LMWH. This result is consistent with recommendations in the relevant guidelines^33^. For safety outcome, although there were no significant differences for major bleeding, CRB and all-cause mortality, patients treated with anticoagulants could have a greater risk for minor bleeding relative to those treated with no anticoagulants, especially in DOACs. Considering the lack of studies on DOACs in pediatric thromboprophylaxis, more clinical evidence is still needed to support the relevant results.

### Meta-analysis of treatment group

For treating thromboembolism, Chen et al.^34^ showed that patients receiving DOACs had low risk for recurrent VTE compared to those receiving SAC, which differs from our results. This may be because our study included a wider range of TEs and included the study of edoxaban. We did not find statistically significant differences in recurrent TEs and repeated imaging outcomes. In addition, we found that there were no statistically significant differences for major bleeding, CRB and minor bleeding, inconsistent with Chen et al.^34^ but similar to Giossi et al.^35^ This may be related to the repeated inclusion of EINSTEIN-Jr trial populations in the former study. Giossi et al.^35^ showed the risk of all-cause mortality caused by DOACs was not inferior to the use of SAC, which consistent with our results. In conclusion, for thrombus treatment, DOACs is not inferior to SAC in terms of efficacy and safety outcomes.

### NMA among anticoagulant therapies

According to the similarity assumption, the NMA of efficacy were classified for prevention and treatment based on the purpose of medication. And the NMA of CRB was conducted in studies covering both prevention and therapeutic population. Six antithrombotic therapies, namely, apixaban, dabigatran, edoxaban, rivaroxaban, SAC, and aspirin were compared to assess the optimal choice for thromboembolic disease in children. Considering the few studies of pediatric thromboprophylaxis, we combined the DOACs for the NMA in prevention group. The ranking showed that DOACs had a low risk for TEs than SAC and aspirin. In the subgroup of meta-analysis, LMWH showed significant advantages, but in NMA, when we included studies that directly or indirectly compared DOACs and SAC, no significant differences were found. Regarding the NMA in treatment group, the risk for TEs associated with these therapies ranked from low to high as follows: rivaroxaban, dabigatran, SAC, and edoxaban. Although there were no significant differences, rivaroxaban and dabigatran showed some advantages in efficacy, which is similar to the data of meta-analyses in adults^36–38^. However, some heterogeneity was revealed in pair-wise comparisons. More clinical evidence is still needed to support the efficacy ranking outcome of antithrombotic therapies in children.

Regarding the safety outcome, the risk for CRB ranked from low to high was: apixaban, edoxaban, dabigatran, SAC, aspirin, and rivaroxaban, consistent with an adult NMA on the risk for bleeding in VTE treatment^39^. In our study, rivaroxaban had a significantly high risk for CRB than that in apixaban. This may be due to fluctuations in the daily peak-trough concentration and drug interactions^39,40^. The NMA was basically consistent with consistency assumption and there was no significant heterogeneity found in global assessment or pair-wise comparisons. As a serious adverse event requiring timely intervention, the ranking result of CRB can provide some guidance for the use of DOACs in children.

Based on the overall assessment of efficacy and safety, and the absence of data on apixaban in TEs, dabigatran may be the best choice for use in children with thromboembolic disease. In addition, the significant advantage of apixaban in terms of safety outcome is expected to make it a new option for children with a high risk of bleeding.

## Highlights

Our review had some strengths. First, we performed a comprehensive search that included a broad pediatric population, involving 2916 patients, and compared more types of DOACs than previous meta-analyses. Second, we divided studies into prevention and treatment groups according to the purpose of medication, which may have improved the evaluation accuracy for efficacy and safety outcomes. Third, to the best of our knowledge, this review was the first to outline available clinical evidence on optimal antithrombotic strategies for children using both meta-analysis and NMA. Our paper summarizes the current best evidence in detail to inform drug selection in future clinical practice and related research.

### Limitations

There were also some limitations to this study. First, considering the few studies of direct comparisons between different DOACs, which may have affected our results, especially in terms of our ranking of antithrombotic therapies. In addition, although we divided studies into prevention and treatment groups, there was still some heterogeneity in our included studies. Finally, because the severity of adverse events other than bleeding was not showed in the included studies. It is difficult to distinguish whether the adverse events are major or minor, so we fail to conducted the other adverse event analysis in this study.

## Conclusion

In the prevention of TEs, the use of anticoagulants had a low risk of TEs and a high risk of minor bleeding compared with no anticoagulants. In the treatment of TEs, the use of DOACs is not inferior to the use of SAC in terms of efficacy and safety outcomes. And according to the overall assessment of efficacy and safety in NMA, dabigatran may be the best choice for children with thromboembolic disease.

### Supplementary Information

Supplementary Information 1.

Supplementary Table S1.

Supplementary Table S2.

Supplementary Table S3.

Supplementary Table S4.

Supplementary Table S5.

## Data Availability

The datasets generated during and/or analyzed during the current study are available from the corresponding author on reasonable request.
