# Supplementary material for: Efficacy and safety of antithrombotic therapy for preventing and treating pediatric thromboembolic disease: a systematic review
Source: Sci Rep. 2024 Jun 11;14:13378. doi: 10.1038/s41598-024-64334-8 (PMC11166992; doi:10.1038/s41598-024-64334-8)
Supplement: Supplementary file 1 — Supplementary Information 1. [file 41598_2024_64334_MOESM1_ESM.pdf]

Supplemental Materials

Efficacy and safety of antithrombotic therapy for preventing and treating pediatric thromboembolic disease: A systematic review

Hongjin Gao[1#], Mingyu Chen[2#], Youqi Huang[2], Huiting Liu[2], Yuze Lin[2], Min Chen[3*]

*Corresponding author: Min Chen

No.134 Dongjie St. Fuzhou 350001, China

Phone: 0086-139-6078-7789

E-mail: chenmin163com@163.com

[1] Department of Pharmacy, Fujian Provincial Hospital, Fuzhou 350001, China. [2] School of Pharmacy, Fujian Medical University. [3] Shengli Clinical College of Fujian Medical University, Fujian Provincial Hospital, Fuzhou 350001, China. These authors contributed equally: Hongjin Gao and Mingyu Chen.[#]

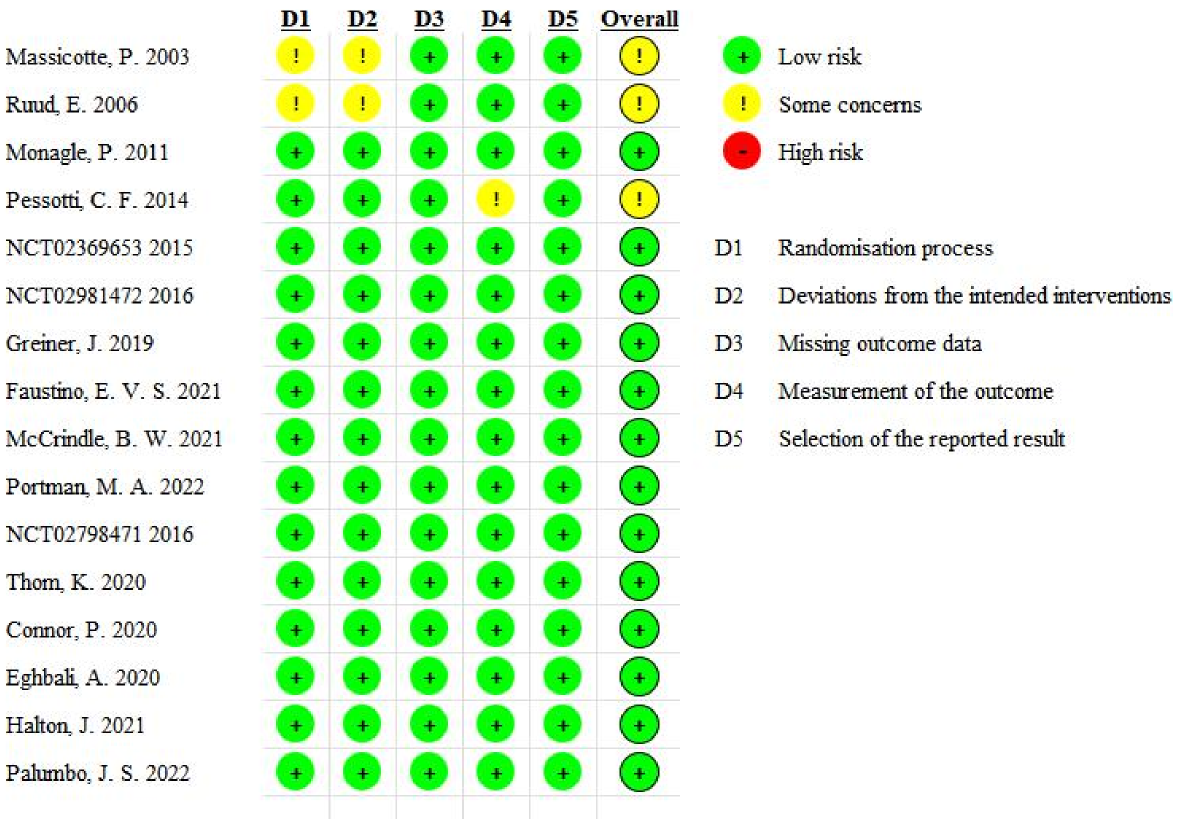

Supplementary Figure S1. Risk of bias summary for included studies which applied intention-to-treat analysis.

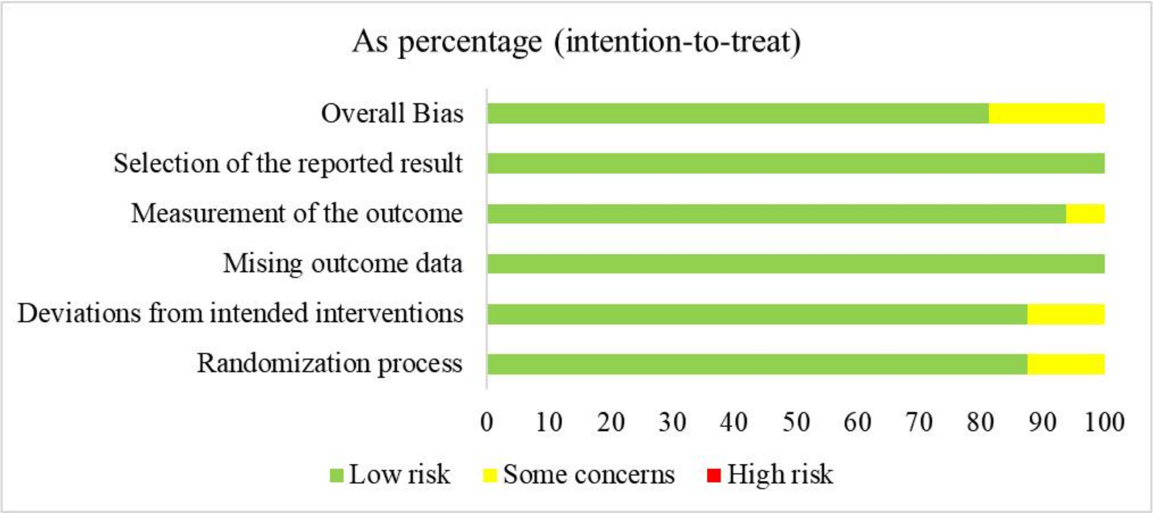

Supplementary Figure S2. Risk of bias summary for included studies which applied intention-to-treat analysis.

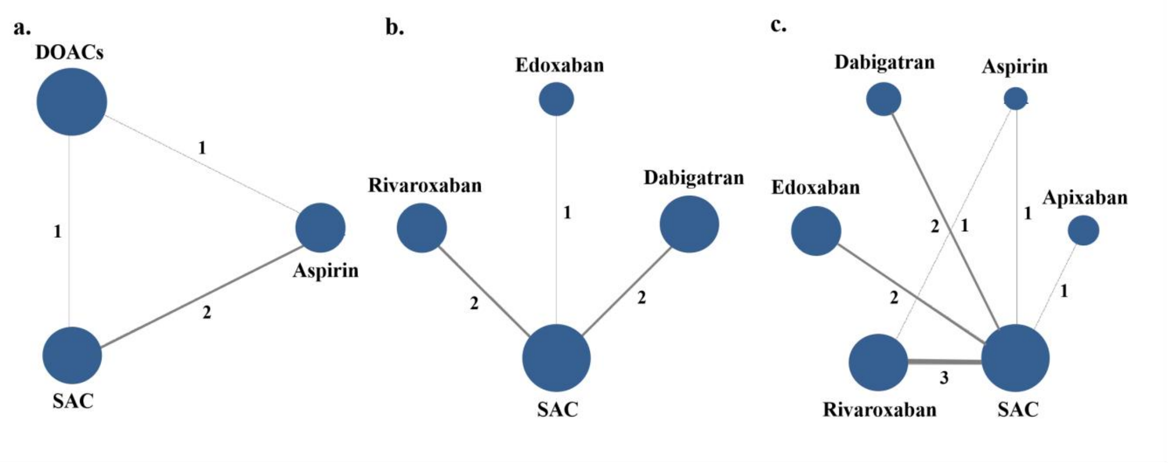

Supplementary Figure S3. Network plot map (a: Thromboembolic events of prevention group; b: Thromboembolic events of treatment group; c: Major or clinically relevant nonmajor bleeding). Note: *DOACs* direct oral anticoagulants, *SAC* standard anticoagulation. The node size and line thickness in the network plot map respectively represent the number of patients included in the corresponding intervention and the number of direct comparison interventions.

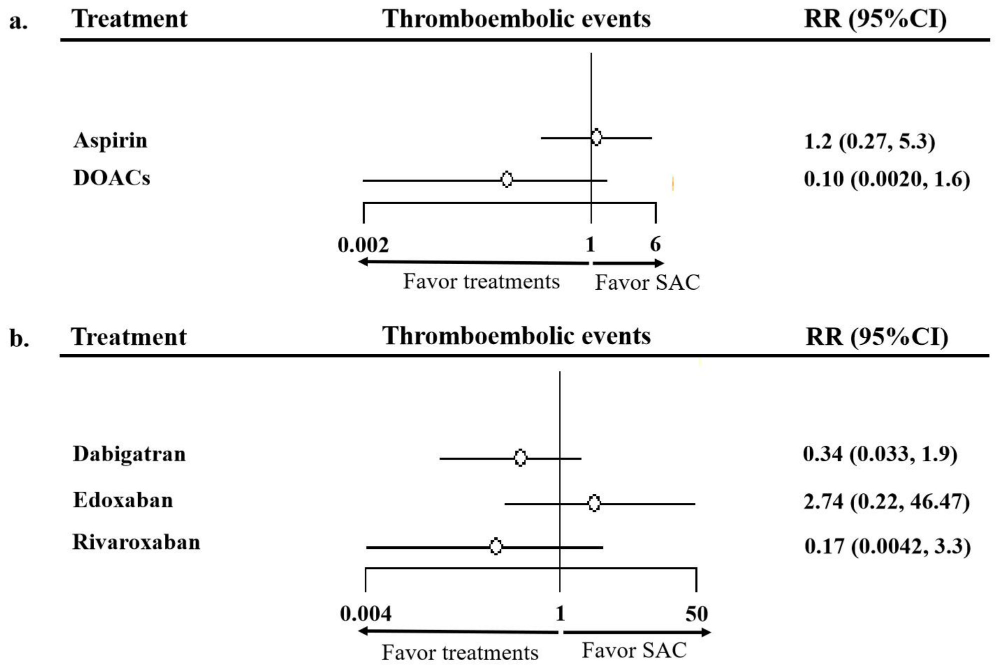

Supplementary Figure S4. Forest plot showing the efficacy outcome (TEs) of others versus SAC in NMA of RCTs (a: Prevention group; b: Treatment group). Note: *TEs* thromboembolic events, *NMA n*etwork meta-analysis, *RCTs* randomized controlled trials, *RR* risk ratio, *CI* confidence interval, *DOACs* direct oral anticoagulants, *SAC* standard anticoagulation.

a.

**Aspirin**		
11.93 (0.9, 495.31)	**DOACs**	
1.2 (0.27, 5.29)	0.10 (0, 1.57)	**SAC**

b.

**Dabigatran**			
0.12 (0, 2.45)	**Edoxaban**		
1.93 (0.05, 110.3)	16.92 (0.33, 1632.19)	**Rivaroxaban**	
0.34 (0.03, 1.9)	2.74 (0.22, 46.47)	0.17 (0, 3.3)	**SAC**

c.

**Apixaban**					
0.04 (0, 1.54)	**Aspirin**				
0.17 (0, 5.49)	3.99 (0.23, 108.24)	**Dabigatran**			
0.24 (0, 6.27)	5.51 (0.29, 128.74)	1.4 (0.08, 17.2)	**Edoxaban**		
**0.03 (0, 0.81)**	0.72 (0.09, 4.38)	0.18 (0.01, 2.31)	0.13 (0.01, 1.65)	**Rivaroxaban**	
0.13 (0, 2.07)	2.98 (0.31, 38.81)	0.76 (0.09, 4.71)	0.55 (0.09, 3.38)	4.13 (0.72, 47.12)	**SAC**

Supplementary Table S6. Head-to-head comparisons table among treatments in network meta-analysis of RCTs (a: Thromboembolic events of prevention group; b: Thromboembolic events of treatment group; c: Major or clinically relevant nonmajor bleeding).

The treatment data are RRs (95% CI) in the column compared to the row. Values less than 1 favor the treatment in the corresponding column, whereas values greater than 1 favor the treatment in the corresponding row. *RCTs* randomized controlled trials, *RR* risk ratio, *CI* confidence interval, *DOACs* direct oral anticoagulants, *SAC* standard anticoagulation.

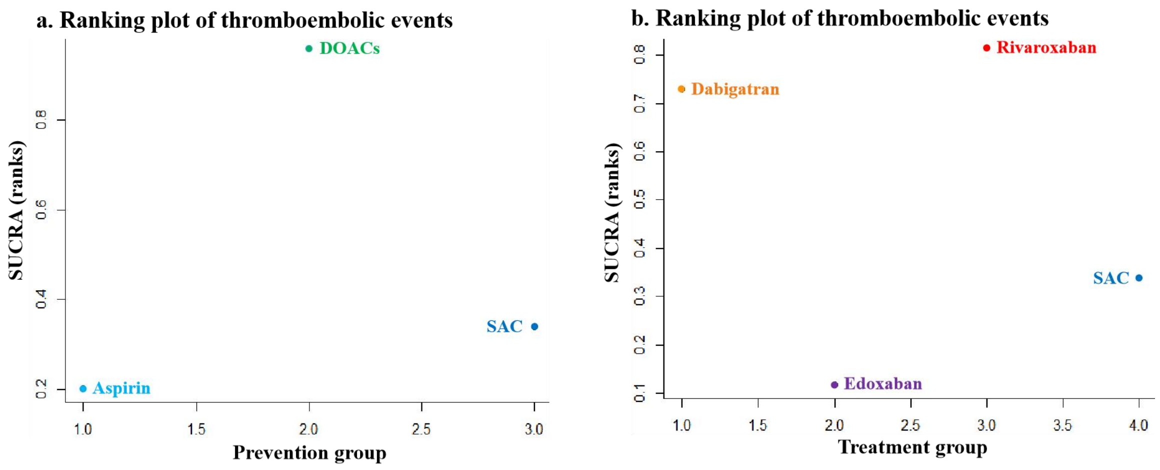

Supplementary Figure S5. Ranking plot for thromboembolic events (a: Prevention group; b: Treatment group). Note: *DOACs* direct oral anticoagulants, *SAC* standard anticoagulation, *SUCRA* surface under the cumulative ranking curves. Higher SUCRA number indicates lower risk of events.

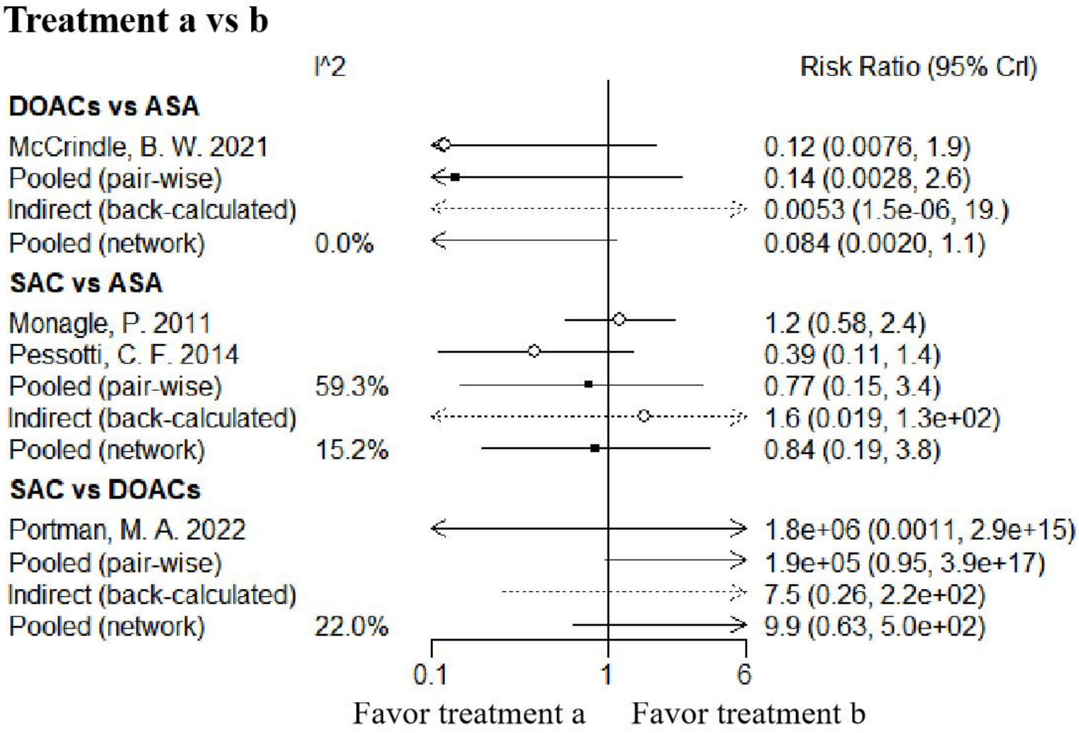

Supplementary Figure S6. Forest plot showing the efficacy outcome (TEs) for the NMA of prevention group. Note: *TEs* thromboembolic events, *DOACs* direct oral anticoagulants, *SAC* standard anticoagulation, *ASA* aspirin.

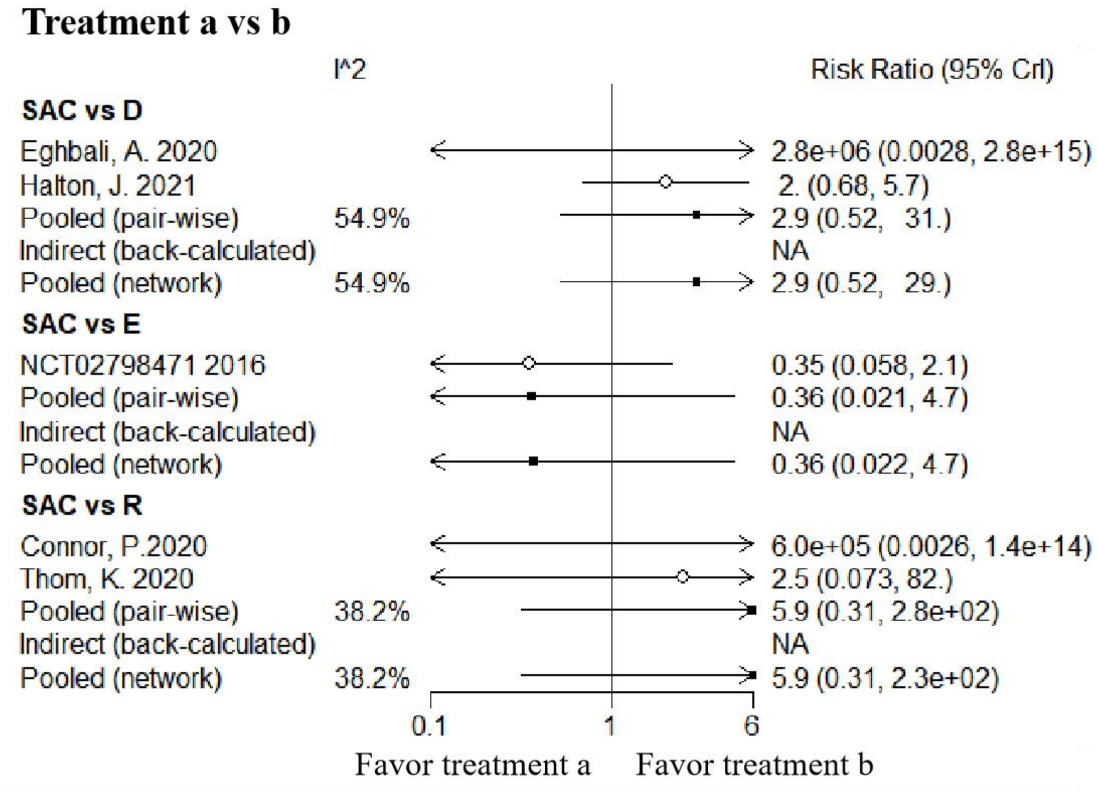

Supplementary Figure S7. Forest plot showing the efficacy outcome (TEs) for the NMA of treatment group. Note: *TEs* thromboembolic events, *SAC* standard anticoagulation, *D* dabigatran, *E* edoxaban, *R* rivaroxaban.

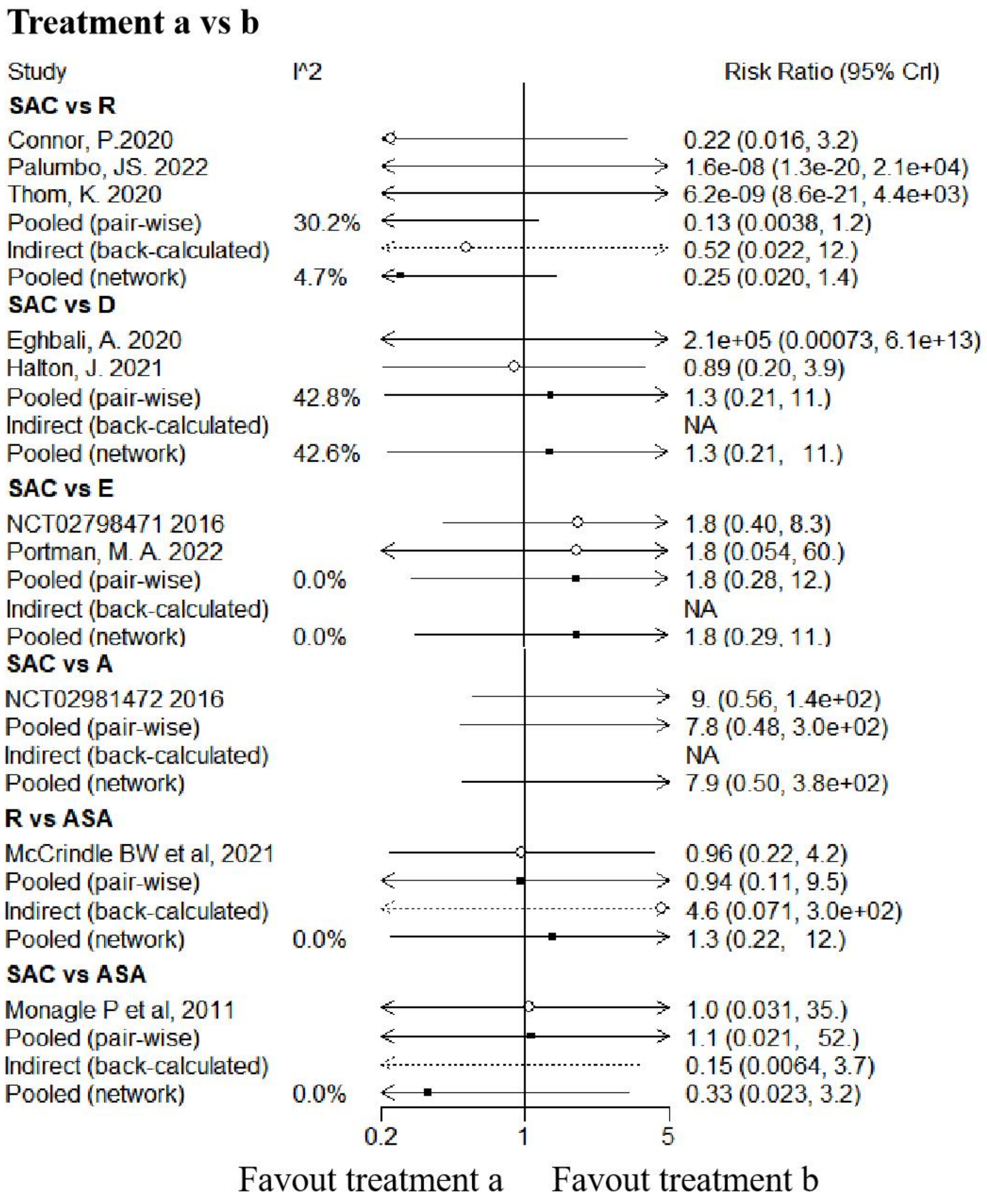

Supplementary Figure S8. Forest plot showing the safety outcome (CRB) for the NMA. Note: *CRB* Major or clinically relevant nonmajor bleeding, *SAC* standard anticoagulation, *R* rivaroxaban, *D* dabigatran, *E* edoxaban, *A* apixaban, *ASA* aspirin.

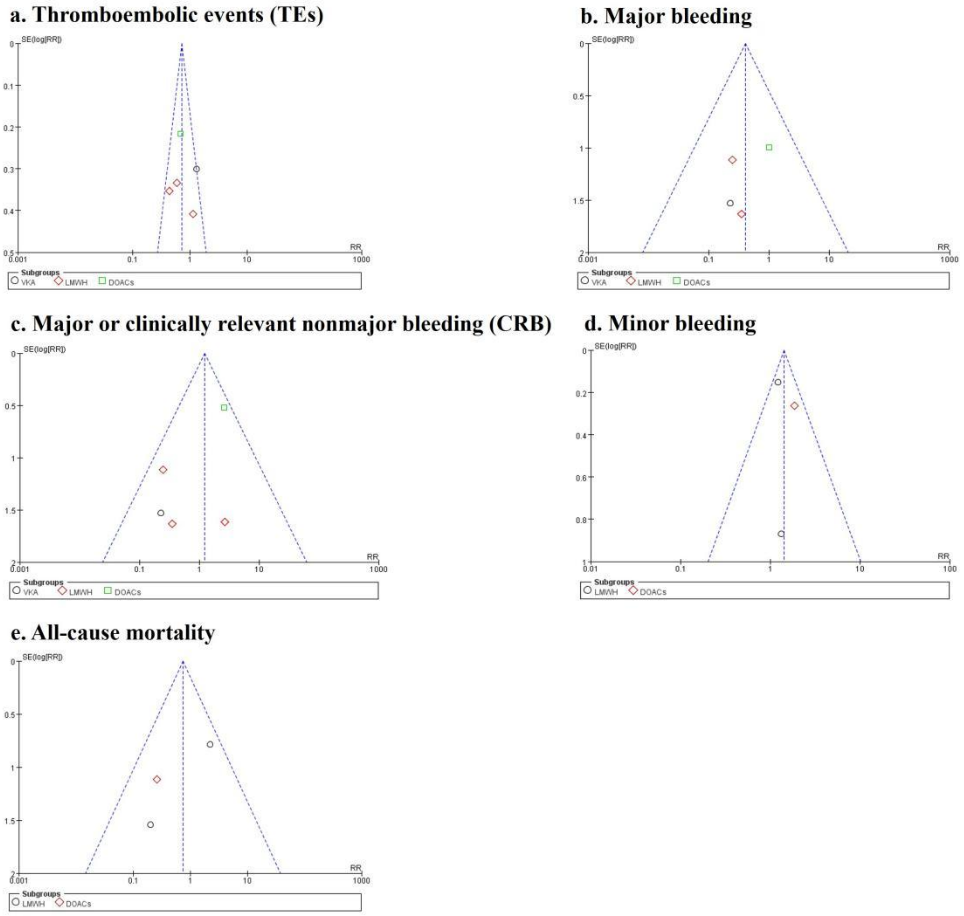

Supplementary Figure S9. Funnel plot of meta-analysis in prevention group (Anticoagulants vs No anticoagulants). *VKA* vitamin K antagonist, *LMWH* low-molecular-weight heparin, *DOACs* direct oral anticoagulants.

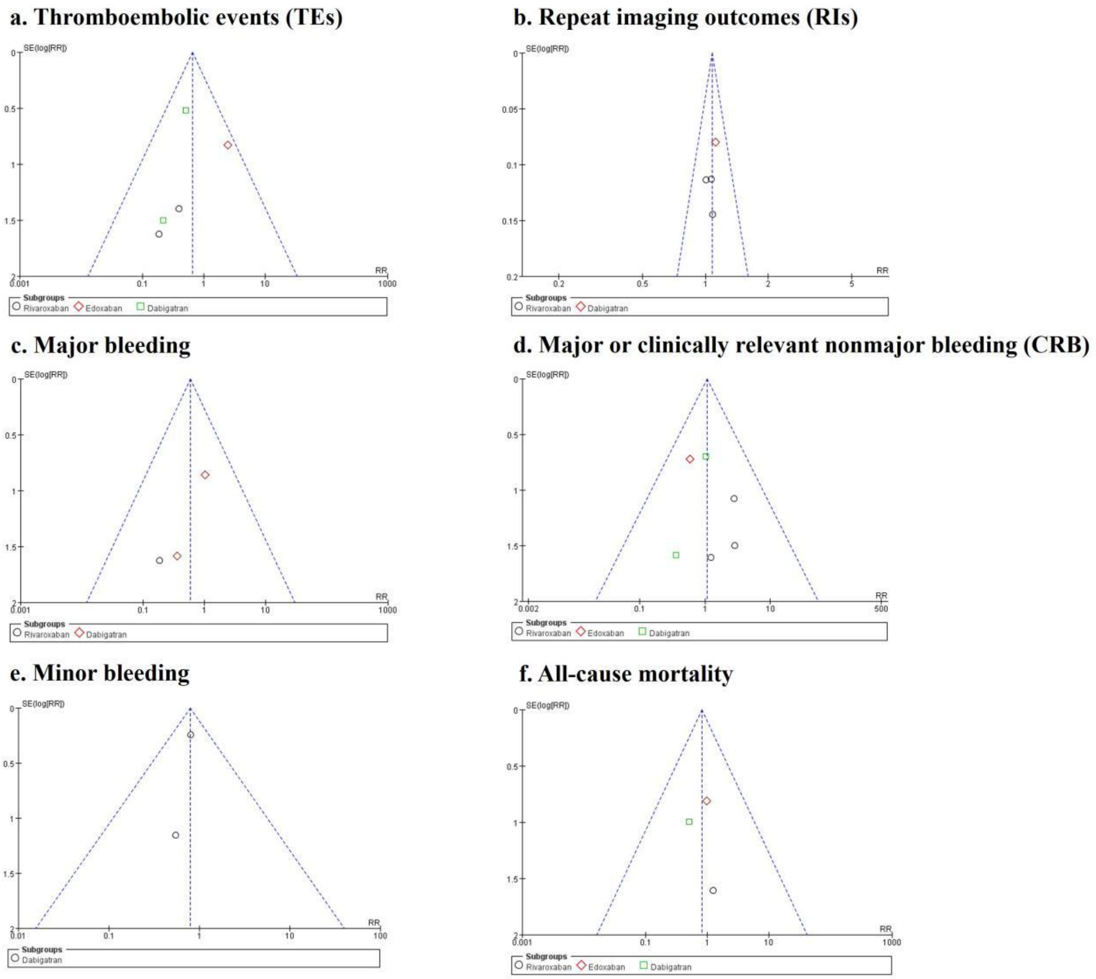

Supplementary Figure S10. Funnel plot of meta-analysis in treatment group (DOACs vs SAC). *DOACs* direct oral anticoagulants, *SAC* standard anticoagulation.
